# Heart Failure: Comorbidities/Multimorbidities

**DOI:** 10.1016/j.jacasi.2025.09.025

**Published:** 2025-12-02

**Authors:** Toru Suzuki

**Affiliations:** University of Leicester, Leicester NIHR Biomedical Research Centre and BHF Centre of Research Excellence, Leicester, United Kingdom and the Institute of Medical Science, The University of Tokyo, Tokyo, Japan

In discussing the complexity of heart failure in the context of concurrent conditions, the definition of comorbidity vs multimorbidity needs to be addressed. Comorbidity is where 2 or more medical conditions are present with a primary condition (eg, heart failure), whereas multimorbidity (also termed multiple long-term conditions) describes 2 or more chronic conditions without any holding priority over the other. Although the term “multiple long-term conditions” is favored in the United Kingdom, where I have a clinical practice as a cardiologist, the term “comorbidity” will be used mainly in the current presentation because the discussion will be centered around the primary condition of heart failure.

The European guidelines^1^ have highlighted both cardiac and noncardiac comorbities, with the latter including diabetes, thyroid disorders, obesity, frailty/cachexia/sarcopenia, iron deficiency/anemia, kidney dysfunction, electrolyte disorders (eg, hypokalemia/hyperkalemia, hyponatremia, hypochloremia), lung disease/sleep-disordered breathing, hyperlipidemia/lipid-modifying therapy, gout/arthritis, erectile dysfunction, depression, and cancer, among other conditions.

The American guidelines,^2^ by contrast, discuss the most common co-occurring chronic conditions among heart failure patients (who are on Medicare). For those <65 years of age, in order of frequency, hypertension, ischemic heart disease, diabetes, hyperlipidemia, anemia, chronic kidney dysfunction, depression, arthritis, chronic obstructive pulmonary disease, and asthma were noted. However, for those ≥65 years of age, although hypertension and ischemic heart disease remained the 2 most prevalent conditions, hyperlipidemia followed by anemia then diabetes were the 5 most common co-occurring conditions.

Much data on the contemporary profile and treatment of heart failure has been accumulated by studies done mostly in Western countries in predominantly Caucasian populations.

One focus of the current presentation is the state of heart failure in Asia.^3^ The prevalence of heart failure is high and increasing, with China, Indonesia, and Malaysia being the 3 countries with highest age-standardized prevalence. The absolute numbers of heart failure prevalence are increasing in Asia overall, with age-standardized prevalence only increasing in Southeast Asia and South Asia. The leading causes of heart failure in Asia and also worldwide are ischemic heart disease and hypertensive heart disease; however, there is more ischemic heart disease compared with hypertensive heart disease in South and Central Asia as well as high-income Asia Pacific countries (eg, Japan, South Korea) compared with East and Southeast Asia, which shows more hypertensive heart disease rather than ischemic heart disease as the cause of heart failure. Rheumatic heart disease is notable in South Asia, and nonrheumatic degenerative mitral valve disease constitutes an important etiology in high-income Asia-Pacific countries and in Central Asia. Mortality from heart failure remains high but varied according to region with 1-year mortality (cardiovascular death as primary cause of death) at a little over 10% for high-income Asia-Pacific countries such as Japan and Korea, whereas Indonesia, Malaysia, and Vietnam have mortality rates exceeding 25%. Further investigations are needed to better clarify the geographical and socioeconomical characteristics of care of patients with heart failure in Asia.

Our group has been investigating newer facets of heart failure pathology such as the contributory role of the gut microbiome,^4^ and have investigated the role of the gut microbiome in the context of multi-morbidity and have developed a clinical risk score to aid in adoption of clinical assessment of the contribution of the gut microbiome to outcomes of heart failure. Age, cardiac markers (history, stage, biomarkers, hemodynamics, medications), renal markers (creatinine), respiratory markers (presence of chronic obstructive pulmonary disease), endocrine disorders (presence of diabetes), along with gut microbiome markers were developed into a calculator for risk stratification of adverse outcomes (mortality and/or rehospitalization).^5^

Patients with heart failure are expected to increase worldwide with aging societies. As a global burden and as a condition that is often associated with comorbidities, further studies will need to explore regional and global practices but also further delineate comorbid conditions which contribute to the complexity of heart failure and its outcomes.

## Funding Support and Author Disclosures

TS was supported by the following funding: the Japan Heart Foundation, National Institute for Health Research (Leicester Biomedical Research Centre), the British Heart Foundation (BHF) including the Centre of Research Excellence Award (RE/24/130031), the Medical Research Council (MRC) UK Consortium on MetAbolic Phenotyping (MAP/UK), Grant-in-Aid for Scientific Research (A) (23H00454) and for Challenging Research (Pioneering) (22K18412) from the Japan Society for the Promotion of Science (JSPS), the LeDucq Foundation, and the Cross-ministerial Strategic Innovation Promotion Program (SIP) on “Integrated Health Care System” Grant Number JPJ012425. The author has reported that he has no relationship relevant to the contents of this paper to disclose.

## References

[1] McDonagh T.A., Metra M., Adamo M., ESC Scientific Document Group (2021). 2021 ESC guidelines for the diagnosis and treatment of acute and chronic heart failure. Eur Heart J.

[2] Heidenreich P.A., Bozkurt B., Aguilar D. (2022). 2022 AHA/ACC/HFSA guideline for the management of heart failure: a report of the American College of Cardiology/American Heart Association Joint Committee on Clinical Practice Guidelines. J Am Coll Cardiol.

[3] Feng J., Zhang Y., Zhang J. (2024). Epidemiology and burden of heart failure in Asia. JACC Asia.

[4] Israr M.Z., Salzano A., Zhan H., Voors A.A., Ng L.L., Suzuki T. (2025). Risk calculator of multimorbid risk of rehospitalisation and death from heart failure - including the contribution of the gut microbiome. Eur J Prev Cardiol.

[5] Suzuki T., Ding H., Yuan F. (2025). Association of gut microbiome biomarkers with mortality in Chinese patients with acute/worsening heart failure. JACC Asia.

